# Role of dendritic cells in spinal cord injury

**DOI:** 10.1111/cns.14593

**Published:** 2024-03-26

**Authors:** Xiaonan Han, Mingkang Zhang, Liyan Yan, Yikun Fu, Hongwei Kou, Chunfeng Shang, Junmin Wang, Hongjian Liu, Chao Jiang, Jian Wang, Tian Cheng

**Affiliations:** ^1^ Department of Orthopaedics The First Affiliated Hospital of Zhengzhou University Zhengzhou Henan China; ^2^ Department of Anatomy, School of Basic Medical Sciences Zhengzhou University Zhengzhou Henan China; ^3^ Department of Neurology The Fifth Affiliated Hospital of Zhengzhou University Zhengzhou Henan China

**Keywords:** dendritic cells, immune regulation, neuroprotection, neurotrophic factors, spinal cord injury

## Abstract

**Background:**

Inflammation can worsen spinal cord injury (SCI), with dendritic cells (DCs) playing a crucial role in the inflammatory response. They mediate T lymphocyte differentiation, activate microglia, and release cytokines like NT‐3. Moreover, DCs can promote neural stem cell survival and guide them toward neuron differentiation, positively impacting SCI outcomes.

**Objective:**

This review aims to summarize the role of DCs in SCI‐related inflammation and identify potential therapeutic targets for treating SCI.

**Methods:**

Literature in PubMed and Web of Science was reviewed using critical terms related to DCs and SCI.

**Results:**

The study indicates that DCs can activate microglia and astrocytes, promote T‐cell differentiation, increase neurotrophin release at the injury site, and subsequently reduce secondary brain injury and enhance functional recovery in the spinal cord.

**Conclusions:**

This review highlights the repair mechanisms of DCs and their potential therapeutic potential for SCI.

## Graphical abstract

Inflammatory response caused by Spinal Cord Injury can do more harm to the injured nervous tissue. Dendritic cells move to the lesion site and regulate inflammatory response by activating T cells and microglia to provide NT‐3 and BDNF, thus provide neuroprotective functions.

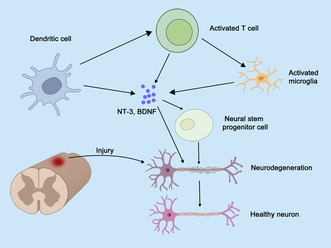

## 1 INTRODUCTION

Spinal cord injury (SCI) is a debilitating condition that results from external forces that damage the integrity or continuity of the spinal cord.^1^, ^2^ SCI can cause a range of physical and psychological effects, including motor, sensory, and sphincter dysfunction below the level of injury, with significant economic consequences for families and society.^3^ Therefore, research into SCI treatment options is crucial to improve patient outcomes.

Over time, researchers have developed new treatment options for SCI. Initially, surgical procedures, traction, and fixation were employed to prevent further damage. Later, hormonal, and neurotrophic treatments were developed to reduce secondary damage. Currently, researchers are exploring neural stem cell transplantation and combination therapy to increase neurologic recovery. However, no highly effective treatment is available to reverse the damage caused by SCI. Instead, the primary objective of treatment is to improve neuroprotection and reduce secondary injury through drug therapy that inhibits the immune response and neuroinflammation, as well as promoting regeneration and repair through cell transplantation and improving the damaged microenvironment.^4^

One crucial area of research is the role of dendritic cells (DCs) in the inflammatory response of SCI. Understanding the function and mechanism of DCs in SCI may help reduce secondary injury and improve overall outcomes. This review aims to examine relevant factors related to the inflammatory response of SCI and the role of DCs in this process.

## 2 LITERATURE SEARCH STRATEGY

To facilitate our analysis, we conducted extensive research using two major scientific databases, PubMed and Web of Science. Our search parameters centered on spinal cord injury, dendritic cells, immune regulation, neurotrophic factors, and neuroprotection. We meticulously examined the articles that pertained to the pathophysiology of SCI, the immune response following SCI, and the influence of neurotrophic factors on SCI. To ensure the accuracy and relevance of our findings, we carefully filtered out outdated and redundant studies.

## 3 PATHOPHYSIOLOGIC CHANGES OF SCI

SCI results from primary and secondary injuries.^5^ Primary injury, also known as mechanical injury, includes spinal cord concussion, contusion, vertebral fracture, damage to spinal cord integrity, axon rupture, cell membrane damage, blood vessel rupture and bleeding, spinal cord tissue compression, and blood supply obstruction. These primary injuries disrupt the balance of the spinal cord environment and increase the permeability of the blood–spinal cord barrier (BSCB).^6^ Primary injury causes secondary injury and involves various mechanisms, such as tissue damage from bleeding, ischemia–reperfusion injury, and microcirculation disturbance caused by local vascular changes. Although the immunoinflammatory response acts as a self‐defense and repair mechanism, an overreaction can harm normal tissue.^7^ Free radicals can cause lipid peroxidation, disrupting the membrane structure, inhibiting metabolism, affecting ion transport, and ultimately leading to ferroptosis.^8^, ^9^ The significant release of glutamate can increase intracellular calcium ion concentration and bind to N‐methyl‐D‐aspartic acid receptors (NMDAR) in neurons and oligodendrocytes, leading to neuronal necrosis or apoptosis, axon demyelination, and conduction block. To sum up, spinal cord injury leads to apoptosis of neuronal cells, microglia, oligodendrocytes, and astrocytes, as well as axon demyelination.^10^, ^11^, ^12^

Following an SCI, four distinct phases of secondary injury take place. These phages are categorized as the acute phase (within 48 h of the injury), the subacute phase (2–14 days) post‐injury, the intermediate phase (14 days–6 months), and finally, the chronic phase (more than 6 months).^13^

During the acute and subacute phases, the body responds to the injury as an emergency, which can lead to further injury. The intermediate and chronic stages are primarily characterized by restructuring dynamic vascular and extracellular matrix molecular deposits and reorganizing local and distal nerve loops. These changes can lead to the formation of cystic cavities and glial scarring, which can, unfortunately, impede the regeneration and connection of damaged nerves.^13^, ^14^ The various pathological changes that can occur due to SCI may cause neuronal cell deaths and the formation of local glial scars, resulting in significant functional losses and challenging recovery.

## 4 THE ROLE OF THE IMMUNE SYSTEM IN SCI

**FIGURE 1:**
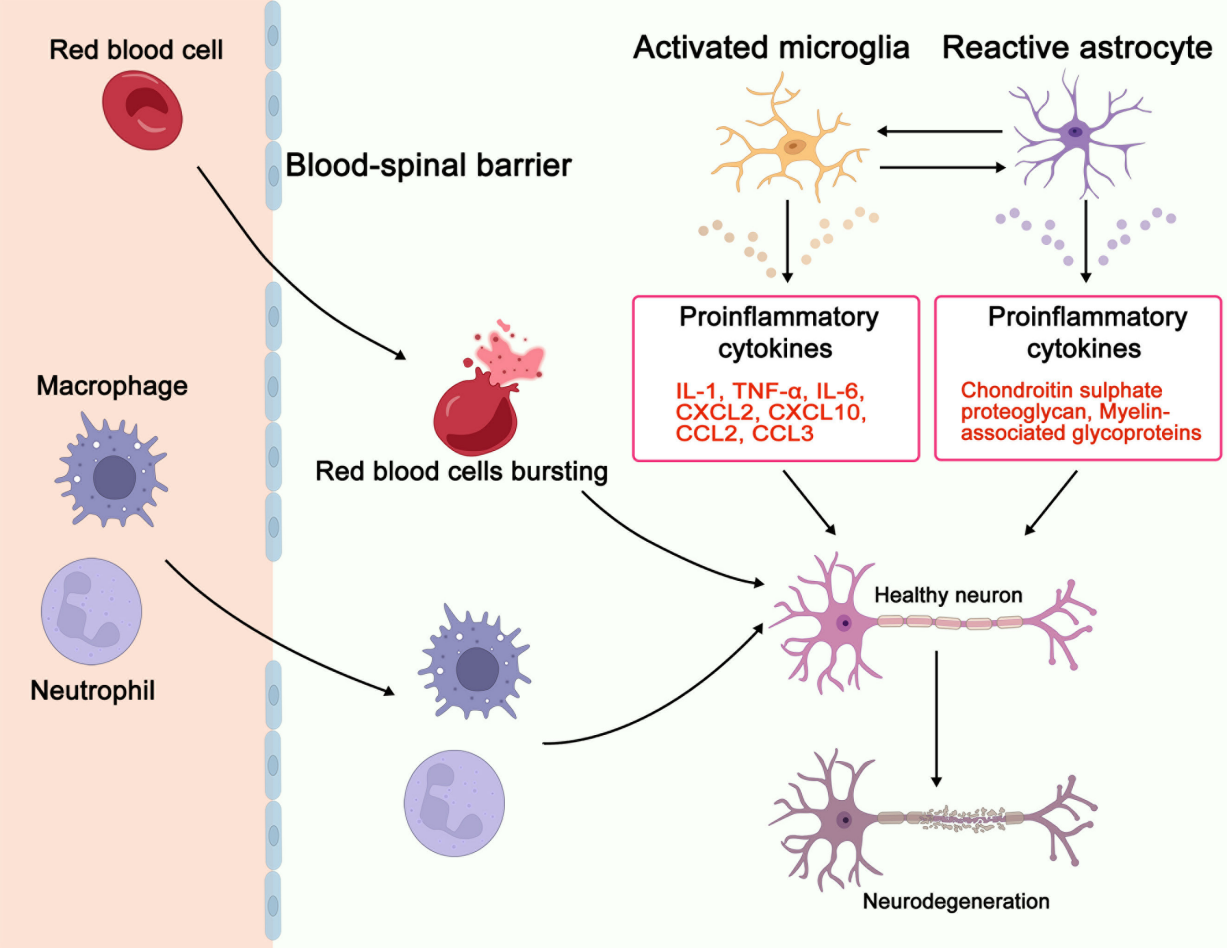
The immune response triggered by spinal cord injury (SCI) during its initial stages. SCI results in the breakdown of the blood–spinal cord barrier, which activates microglia in the damaged tissue. These microglia then release pro‐inflammatory cytokines, which stimulate the migration of neutrophils and macrophages to the injury site. The collaboration of these inflammatory cells causes additional damage to healthy neurons in the area, leading to what is considered the second injury.

SCI elicits a multifaceted response from the human body, primarily influenced by activated glial cells in the central nervous system (CNS) and immune cells originating from the peripheral system. These cells release many substances, including cytokines, chemokines, reactive oxygen species, and second messengers, which are crucial in responding to SCI. The outcome of this complex response is determined by the interplay of immune, biochemical, and pathological reactions, with the severity of injury contingent upon the primary injury mechanism and the pathological processes of the secondary injury. Please refer to Figure 1 for a visual representation of the primary inflammatory cells and cytokines manifesting after SCI (Figure 1).

### 4.1 Cellular immune response

SCI can disrupt the BSCB, exposing CNS antigens to the spinal cord and lymphoid tissues throughout the body. This exposure triggers an inflammatory response that involves various immune cells, such as neutrophils, monocytes, lymphocytes, and microglia.^15^

Microglia are immune cells that primarily reside in the CNS and function as phagocytes. Typically, they assist in regulating the microenvironment of the CNS. However, when brain tissue is damaged, microglia can be triggered by various molecules related to injury, cell fragments, myelin sheath, or γ‐interferon.^16^ Within 2–3 days after injury, microglia migrate to the affected area of the spinal cord and release cytokines, including IL‐1, TNF‐α, and IL‐6. In addition, they express a range of chemokines such as CXCL2, CXCL10, CCL2, and CCL3, which initiate a neurotoxic cascade that leads to apoptosis and necrosis of endothelial cells, neurons, axons, and oligodendrocytes. This cascade culminates in secondary SCI and exacerbates the local inflammatory response.^17^

Astrocytes, a type of glial cell, play a vital role in the CNS by maintaining the structure and function of the blood–brain barrier. They provide nutrients and growth factors and remove excess fluids, ions, and neurotransmitters, such as glutamate, from the extracellular microenvironment.^18^ Their intricate communication network involves interaction with neurons through synapses or cell connections and releasing signal molecules to communicate with neighboring cells.^19^ However, astrocytes can undergo hypertrophy, migration, and proliferation during inflammatory triggers, leading to glial scars that prevent axon regeneration. Activated astrocytes secrete inhibitors, such as chondroitin sulfate proteoglycan, myelin‐associated glycoproteins, and oligodendrocyte myelin glycoproteins, that impede axon regeneration.^20^ Furthermore, overstimulation of niacinamide adenine dinucleotide phosphate oxidase (NOX) in astrocytes can lead to glutathione depletion, a potent antioxidant. This depletion results in the overproduction of glutamate neurotoxin, which damages neurons and oligodendrocytes, ultimately leading to neuronal dysfunction.^21^

Initially, neutrophils assemble at the site of injury for a brief period. Subsequently, antigen‐specific T lymphocytes (T‐LC) and nonantigen‐specific macrophages (MC), orchestrate the onset of secondary autoimmunity.

After an SCI, the immune cells that respond to the injury include neutrophils originating from the blood. Within 3–6 h of the injury, the levels of neutrophils rapidly increase and reach their peak within 24 h.^22^ Blood vessels surrounding the injury site express selectin P and selectin E, which help the attachment of neutrophils to vascular endothelial cells. They subsequently migrate to the injury site under the influence of chemokines to participate in the local inflammatory response. Neutrophils promote histiocytic proliferation and wound repair while removing necrotic tissue.^23^ Still, the process is associated with the release of chemokines and cytokines, which may lead to necrosis and apoptosis of neurons and oligodendrocytes, thereby hindering the recovery of nerve function after injury.^22^ Immediately after acute trauma, monocytes infiltrate the injury site and differentiate into macrophages. Activated macrophages and microglia are identical in morphology and immunohistology.^24^ Activated macrophages and microglia can phagocytose damaged cells and myelin fragments and secrete various cytokines, growth factors, and free radicals, possibly leading to further damage.^25^

The extent and timing of T lymphocyte infiltration differ among different species. In rats, T cells gradually infiltrate the injured spinal cord, with infiltration beginning 12 h post‐injury. The numbers of these cells peak on the 7th day, after which they decline by 50% over the following 3 weeks. Conversely, in mice, T cells enter the injury site significantly only 14 days after the injury. Their numbers increase twofold between the 2nd and 6th weeks and remain active for extended periods in mice with SCI.^26^, ^27^, ^28^

The activation of T cells is triggered by specific surface receptors that identify antigens and initiate autoimmune responses.^29^, ^30^ Compared to innate immune responses, autoreactive T cells can cause direct harm to neurons and glial cells and indirectly affect neuronal function and survival through the release of pro‐inflammatory cytokines (IFN‐γ, IL‐1β, TNF‐α, IL‐12) and chemokines (CCL2 and CCL5, among others).^31^

CD4 + T cells are categorized into effector (Teff) and regulatory (Treg) T cells, which work together to maintain immune balance.^32^ During SCI, the activity of Teff cells increases, disrupting the Treg‐Teff balance. Consequently, pro‐inflammatory cytokines and chemokines are produced, promoting tissue damage and neuronal and oligodendrocyte apoptosis.^33^ B lymphocytes also contribute to the inflammatory response,^34^ with antibodies specific for myelin basic protein (MBP) and other CNS antigens released after SCI.^35^

### 4.2 Molecular immune response

Examining cytokines and other inflammatory mediators released during the inflammatory response associated with SCI is of utmost importance. Chemokines, which attract immune cells during infections, inflammation, and CNS trauma, are critical to the study.^36^ Chemokines have two types^37^: the α family (CXC), which attracts polymorphic cells, and the β family (C‐C), which sends signals to macrophages, lymphocytes, and granulocytes. CXCL10 and CCL2 mRNA are present in the spinal cord under normal circumstances, with CXCL10 mRNA present in low levels. However, after SCI, the expression of CXCL10 mRNA increases significantly within an hour and peaks at 6 h. The expression continues to rise until the 5th day and returns to its initial level by the 14th day.

On the other hand, CCL2 mRNA expression peaks after 24 h and returns to a low level after 14 days,^36^ with the increase occurring an hour after SCI. Recent studies have indicated that CXCL10 inhibits endothelial cell angiogenesis and growth through the CXCR3B receptor. CXCL10 is not conducive to recovery from SCI, and reducing its expression after injury may help reduce adverse reactions.^38^, ^39^

After an SCI, the pro‐inflammatory cytokine TNF‐α induces inflammation. Its expression is highest in the hours after the injury. TNF has various biological functions that can exacerbate tissue damage and trigger autoinflammation. The primary impact of TNF on post‐SCI inflammation can be summarized as follows^40^: (1) It increases nitric oxide synthase activity, which generates more nitric oxide, intensifying cytotoxicity and collaboratively increasing glutamate‐mediated cell death. (2) It amplifies the chemotaxis of inflammatory cells, resulting in a more robust inflammatory response. (3) It affects vascular endothelial cell physiology, causing increased vascular permeability and prompting the release of inflammatory cells and mediators.

Nitric oxide (NO) is a molecule produced by nitric oxide synthase (NOS) that plays a vital role in immune regulation and other bodily reactions. There are three subtypes of NOS: neuronal NOS (nNOS, NOS‐1), inducible NOS (iNOS, NOS‐2), and endothelial NOS (eNOS, NOS‐3).^41^ Among these, iNOS generates the highest amount of NO and contributes to the inflammatory process after SCI. Inflammatory cytokines such as TNF‐α and IL‐1, along with glycosphingolipids, can activate iNOS production in glial cells and macrophages, leading to an inflammatory response.^42^ The NO produced by iNO can cause microglia‐mediated demyelination and neuronal apoptosis.

Interleukins (ILs) are essential substances white blood cells produce that play multiple biological roles. They regulate the immune response, stress response, and inflammation. In SCI, different types of ILs have various functions. The pro‐inflammatory Ils, including IL‐1, IL‐2, IL‐6, IL‐11, and IL‐15, and the anti‐inflammatory ILs, such as IL‐4, IL‐10, and IL‐13, interact to create a complex network that worsens the damage, known as the “waterfall effect”.^43^, ^44^

In spinal cord ischemia–reperfusion injury, IL‐1β expression increases, and it works with other factors, such as NF‐κB, TNF‐α, and IFN‐γ, to control the expression of adhesion molecules. This cytokine storm causes post‐injury inflammation and secondary injury.^45^ Meanwhile, IL‐8 induces chemotaxis of neutrophils and T lymphocytes, altering the physiological structure of inflammatory cells, increasing their adhesion to vascular endothelial cells, and triggering cell deformation, degranulation reactions, respiratory bursts, and lysosomal enzyme release. All of which accelerates the activation of inflammatory cells.^46^ However, IL‐10 is believed to inhibit early inflammation after SCI by preventing the activation of pro‐inflammatory cells.^47^ In summary, ILs are critical in regulating the immune system and responding to inflammation in SCI. A better understanding of their functions can help us comprehend the mechanisms underlying SCI, leading to effective treatments.

According to research, NF‐κB is crucial in regulating cell survival, apoptosis, proliferation, and differentiation.^48^ Following SCI, producing various oxygen radicals and cytokines can activate NF‐κB in nerve cells, microvascular endothelial cells, and glial cells. Upon entering the nucleus, it binds to the target sequence, regulating the transcriptional activity of genes related to intercellular adhesion molecule‐1 (ICAM‐1), TNF‐α, IL‐1, IL‐6, and others. This sequence of events forms a positive feedback loop, thereby increasing the inflammatory response in the affected area. As a result, the area of tissue damage increases, and nerve and synapse regeneration is impeded.^49^ However, recent research suggests that NF‐κB has a dual role in the inflammatory response of SCI. It can also promote neuroprotection and synaptic repair by increasing the levels of antiapoptotic proteins and by inducing nuclear transcription factors.^50^

The occurrence of an SCI leads to immune‐inflammatory responses that can be detrimental to the injury site without appropriate intervention. However, some cells and mediators involved in the inflammatory response possess reparative properties. For example, the secretion of anti‐inflammatory cytokines by IL‐10 converts microglial cells into an anti‐inflammatory phenotype,^51^, ^52^ which protects neurons and axons.^53^ Treg cells exert inhibitory effects on the activated Teff cells while inhibiting monocyte/macrophage inflammation in the peripheral immune system by producing IL‐10 or recruiting CD4 + T cells to secrete IL‐10. Furthermore, Treg cells reduce the production of cytokines, chemokines, cell adhesion molecules, and reactive oxygen species. By limiting damage caused by monocyte/macrophage inflammation in the spinal cord, Treg cells have the potential to promote repair.

Secondary damage can occur due to an excessive expression of pro‐inflammatory components or an imbalance between pro‐inflammatory and anti‐inflammatory effects. Empirical studies have shown that a well‐regulated immune response can protect the CNS during diseased conditions.^54^ Rossignol et al.^55^ suggested regulating the intensity and timing of the inflammatory response to enhance spinal cord healing and prevent further damage. It is crucial to consider the timing of the injury and the nature of spinal cord immunity, as different periods can have opposite effects on nerve damage and protection.

It is essential to note that DCs have multiple functions beyond their role in presenting antigens and activating T cells. Research shows that DCs can contribute to tissue repair and regeneration.^56^, ^57^ As a potential therapy for SCI, the efficacy of the injection of DC has been examined. However, further research is necessary to determine how DCs can aid tissue repair and regeneration after SCI.

## 5 THE ROLE OF DCs in SCI

### 5.1 Introduction of DCs

DCs are a type of immune cells that exist throughout various tissues and organs in the body.^58^ They are derived from the precursor cells of the bone marrow's myeloid and lymphoid precursor cells. Initially, immature DCs can be found around interstitial capillaries in parenchymal organs. These cells can engulf exogenous antigens, which are then processed and presented to T cells. Once mature, they migrate to lymphatic organs, activating naive T cells and transforming them into effector T cells. These activated T cells initiate and sustain immune responses.^59^, ^60^

DCs are high‐potent antigen‐presenting cells (APCs) with an antigen‐presenting capacity that exceeds other APCs by more than 1000 times.^61^ This unique feature enables DCs to activate naive T cells and initiate immune responses against viruses, bacteria, and tumors. DCs are instrumental in forming and shaping immune responses by presenting antigens to T cells.

DCs process antigens into antigenic peptide–MHC complexes on the cell surface and interact with T cell receptors. Co‐stimulatory signals, including CD40, CD80, CD86, PDL‐1, PDL‐2, and CD54, provide the necessary T‐cell activation signals. The third signal determines the type of effector cells into which naive T cells differentiate. IL‐12, IL‐18b, and IFN‐γ lead to differentiation in Th1 cells, while OX40 initiates differentiation in Th2 cells. High levels of ICOSL expression and IL‐10 secretion lead to Treg cell production.^62^, ^63^

DCs can be divided into two categories: myeloid DCs (MDCs) and lymphoid DCs (LDCs), depending on their origin.^64^ MDCs are produced from myeloid stem cells, stimulated by granulocyte‐macrophage colony‐stimulating factor (GM‐CSF). On the other hand, LDCs are derived from lymphoid stem cells and are generated by cytokines like IL‐3H and CD40L. DCs can also be classified into conventional DCs (cDCs) and plasmacytoid DCs (pDCs) based on their transcription factors and functions, while monocyte‐derived DCs (moDCs) are also included.

The cDCs can be further divided into subgroups: cDC1 and cDC2. cDC1 cells activate CD8^+^ T cell activation and proliferation by cross‐presenting exogenous antigens through Class I molecules of the major tissue phase compatibility complex (MHC). In contrast, cDC2 cells can induce T helper type 1 (Th1) and Th17 responses after activation^65^, ^66^ (Table 1).

**TABLE 1:** Dendritic cell subsets, locations, surface markers, and function.

Subsets	Locations	Surface markers	Function
cDC1s	Blood, Skin, Liver, Lungs, Intestines, Spleen, Lymph nodes	XCR1, CLEC9A, CADM1, CD8α, CD103, CD11c, MHC II	cDC1s present antigens through MHC class I molecules that activate CD8^+^ T cells
cDC2s	Blood, Spleen, Kidney, Dermis, Intestine	CD11b, SIRPα, TLR4, TLR5, CD4, CD11c, MHC II	cDC2s present antigens through MHC class II molecules and activate CD4^+^ T cells
pDCs	Blood, Lymph nodes, Tonsils	CD123, CD303, CD304, CD62, CD45RA, MHC II, TLR7, TLR9	pDCs produce a large amount of IFN I/α, participate in antigen cross‐representation, and exert a strong antiviral immune response
Inf DCs	Inflammation tissue	MHC II, CD11c, CD206, CD1b, CD1c	Inf DCs are associated with atopic dermatitis, psoriasis, and rheumatoid arthritis and play a critical role in microbial infections

Abbreviations: CADM1, cell adhesion molecule 1; cDC1s, conventional dendritic cells 1; cDC2s, conventional dendritic cells 2; CLEC9A, C type lectin receptor 9A; Inf DCs, inflammatory DC; MHC II, major histocompatibility complex II; pDCs, Plasmacytoid dendritic cells; SIRPα, signal regulatory protein alpha; TLR4, toll‐like receptors 4; TLR5, toll‐like receptors 5; TLR7, toll‐like receptors 7; TLR9, toll‐like receptors 9; XCR1, Chemokine Receptor 1.

DCs play a critical role in regulating immune responses by presenting antigens.^67^ They inhibit T cell proliferation by secreting IL‐4, IL‐10, and IL‐5 in the later stages of the inflammatory response.^68^ Immature DCs lack the signal to induce T cell activation, resulting in T cells showing immune tolerance. Furthermore, immature DCs can produce high levels of NO, which can cause apoptosis of autoreactive T cells and DCs themselves, thus interrupting the antigen presentation process.^69^

### 5.2 Functions of DCs in SCI

Research studies have determined that DCs are present in the regular meninges, the choroid plexus, and the cerebrospinal fluid (CSF); however, they are not present in the normal brain parenchyma.^70^ Under physiological conditions, DCs in the meninges and choroid plexus are immature and incapable of presenting antigens. Nevertheless, these cells can absorb CSF to remove self‐proteins in their environment. Subsequently, the DCs can migrate to the T cell region through the pathway of CSF‐olfactory nerve‐ deep cervical lymph node. Upon reaching the lymph nodes, inactive T cells eliminate them, contributing to peripheral immune surveillance and tolerance.^68^

In abnormal immune responses in the CNS, DCs play an essential role in activating Th cells.^71^ DCs capture autoantigens and migrate to lymphoid tissues upon activating pathogens and necrotic tissues. They exhibit multiple surface markers and cytokines, including IL‐6 and IL‐12, activating reactive T lymphocytes.^72^ These activated T cells can cross the blood–brain barrier, enter the immune‐privileged organ, and initiate an immune response. In specific pathologic processes, such as MS, DCs can contribute to the chronic progression of the disease by activating lymphocytes in situ. The differentiation of naive T cells into Th1 or Th2 cells depends on several factors, such as maturity, number, surface factors, and the DCs' environment. Th1 cells produce IFN‐γ, IL‐2, and TNF‐β, leading to enhanced cell‐mediated immune responses, while Th2 cells produce IL‐4, IL‐5, and IL‐13, leading to improved humoral immunity.^73^

The location of an SCI has been shown to experience an increase in DCs.^74^ However, much remains to be discovered about their origin within the CNS. It is believed that DCs may infiltrate the brain parenchyma through various pathways during inflammation:

1. One such pathway involves immature DCs attracted to CXCL12 and CCL20 secreted by astrocytes in the affected region. These cells travel from the internal carotid to the post‐capillary micro‐veins before leaving the blood vessels and reaching the lesion site to contain antigens. Notably, these DCs remain within the vascular sheath until they mature into MDCs after leaving the blood vessels.^70^
2. Specific chemokines such as XCLI2, CCL2, CCL4, CCL5, and C5a are expressed at the injury site during pathologic conditions. These chemokines can attract immature DCs from the CSF, meninges, and choroid plexus. XCL12 and GM‐CSF are particularly important in this process as they help immature DCs mature into fully functional DCs. This process happens when the DCs cross the inflammatory BSCB and reach the lesion site.^75^
3. Studies have shown that glial cells can transform into DCs in vitro brain tissue culture, transplantation immunity, and experimental stroke. These DCs exhibit the typical MDC phenotype and express CD11b, a unique glial cell marker. This finding suggests that activated glial cells produce DCs in the CNS. However, further investigation is required to determine the exact origin and mechanisms behind the production of DCs in the CNS.

Recent studies indicate that DCs may have potential neuroprotective effects in SCI cases. Notably, the research by Yaguchi et al. has shown that DCs can potentially treat SCI in marmosets.^76^ Further investigation has indicated that T cells may significantly influence DCs' neuroprotective effects. The spinal cord releases various autoantigens following an SCI, stimulating immature DCs and prompting them to mature into fully functional DCs.^77^ These cells then travel to draining lymph nodes and activate CNS‐specific T cells, leading to a Th1 (Th1/Th0) bias. Antigen‐specific T cells subsequently cross the blood–brain barrier and accumulate at the site of CNS lesions,^78^ producing cytokines.

At an early stage, DCs cannot present antigens and require ample time to establish adaptive immunity specific to the CNS. This fact implies that T cells activated by native DCs or locally injected inactivated DCs may not be able to provide neuroprotection during the early stage of injury. Studies have shown that DC pulse vaccination with CNS‐associated myelin antigens can reduce secondary damage in rat and mouse models of spinal cord contusion. Additionally, passive immunity mediated by A91‐DC has been shown to produce significant therapeutic effects in SCI mice.^79^ Some studies have suggested that only auto‐specific CNS antigens produced in the passive immune response mediated by DCs have therapeutic effects on SCI. Compared to a single specific antigen or peptide, whole proteins may incorporate multiple immunogenic epitopes and possibly more beneficial autoantigens in a homogenate preparation and are more likely to contain class I and class II‐restricted epitopes. Vaccination with homogenate proteins has been shown to promote axon regeneration and recovery from SCI.^80^

**FIGURE 2:**
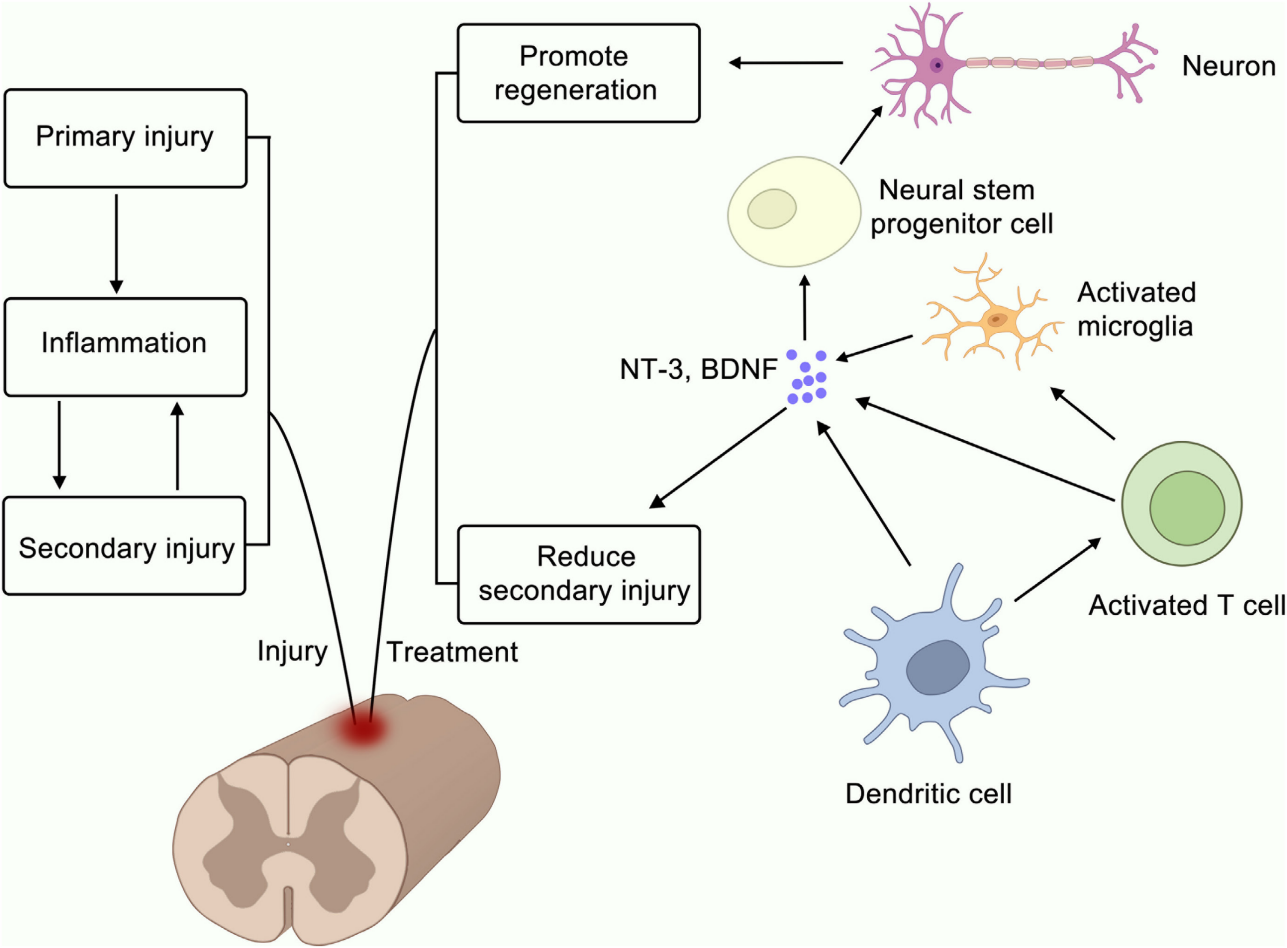
Illustration of how dendritic cells (DCs) could potentially treat spinal cord injury (SCI). The mechanism involves DCs activating T cells, which then interact with microglia to trigger the secretion of neuroprotective factors. This ultimately leads to the promotion of neuron regeneration and a decrease in secondary injury.

Moreover, passive immunity mediated by hpDCs can significantly reduce the barrier effect of glial scars on nerve regeneration and enhance nerve functions in mice.^81^ Studies have also shown that hpDCs can efficiently activate T cells to proliferate in large numbers in vitro, and intraperitoneally injected hpDCs can migrate to the injury site in mice and promote the production of neurotrophic factors.^82^ Activated T cells release more IFN‐γ, a Th1 cytokine^83^ that has a role in neuroprotection despite its association with neurodegeneration and tissue destruction.^84^ Activated T cells also produce neurotrophic factors such as brain‐derived neurotrophic factor (BDNF), nerve growth factor, NT‐3, and NT‐4/5, continuously released at the lesion site.^85^ T cells induce resident microglia or astrocytes to produce neurotrophic factors by secreting IFN‐γ.^86^ Additionally, T cells can enhance the glutamate clearance capacity of astrocytes, suggesting their protective effect on a damaged CNS.^87^ Research has indicated that DCs can produce neurotrophins, including nerve growth factor, BDNF, neurotrophin (NT)‐3, and NT‐4/5, which can minimize neuronal loss and axonal damage, resulting in a neuroprotective effect upon relocation to SCI^88^ (Figure 2).

Neurotrophins are a group of proteins that support the survival of neurons by receptor‐mediated processes. These proteins play a crucial role in maintaining the normal functioning and response of the nervous system, especially in the case of trauma. BDNF and NT‐3 are essential neurotrophic factors that help to repair damage and protect the nervous system. BDNF has been shown to reduce NO and lipid peroxide at the injury site.^89^ This protein activates the TrkB receptor on the neuronal membrane and initiates downstream gene transcription via the cAMP response element binding protein (CREB).^90^, ^91^ In addition, the BDNF–TrkB pathway is critical in neuroprotection, regulation of inflammatory factors, synaptic plasticity, and controlling spasms and pain after CNS injury.^92^ Recent research has shown that rats with a blocked BDNF–TrkB signaling pathway after SCI demonstrated significantly lower motor function recovery compared to the unblocked group.^91^ Similarly, Ying et al.^93^ found that treadmill training (TT) increases the BDNF/TrkB‐CREB signaling pathway and protects the BSCB after SCI.

NT‐3 has exhibited significant potential in adding to the recovery of damaged nerve cells. NT‐3 promotes the growth, development, differentiation, and maturation of neurons while also safeguarding them from injury. It prevents the death of motor neurons during embryonic and postnatal stages, reduces the death rate of injured spinal cord neurons, and enhances the survival of damaged spinal cord neurons. Furthermore, NT‐3 stimulates the regeneration of nerve cell axons, encourages axon elongation, and facilitates the myelination of regenerated axons. NT‐3 also supports the growth and regeneration of corticospinal tract fibers, sensory conduction fibers of the dorsal column, and dorsal root ganglion fibers after SCI. It selectively enhances the survival and growth of spinal neurons. It prevents apoptosis of cortical motor neurons, red nucleus, dorsal nucleus, and spinal sensory projection neurons after axonal disconnection, promoting their survival.^94^, ^95^, ^96^ Recent studies have demonstrated that NT‐3 promotes nerve fiber and myelin sheath regeneration after injury through the TrKC‐mediated PI3K/AKT/mTOR and PI3K/AKT/CREB pathways.^97^ Research on canids^98^ and nonhuman primates^99^ has revealed that NT‐3 can create a conducive microenvironment for regeneration and facilitate endogenous repair after severe SCI. Moreover, NT‐3 plays a vital role in preventing the death of central neurons.^100^, ^101^

Wang et al.^102^ conducted a study using adult mice with T10 moderate contusion to investigate the potential effects of reverse transporting NT‐3 to the lumbar spine. Their findings demonstrated a significant decrease in spinal motor neuron dendrite atrophy caused by SCI, resulting in enhanced motor recovery. Han et al. subsequently validated that NT‐3 could foster functional recovery of thoracic cord motor neurons at the rostral level by stimulating the neural circuit of the spinal‐lumbar motor neuron.^103^

These effects can regulate the microenvironment to promote the survival of neural stem progenitor cells (NSPCs). NSPCs are versatile stem cells that can respond to nervous system injuries and diseases and possess the potential for self‐regeneration and multi‐differentiation. In vitro, NSPCs can differentiate into three types of nerve cells: neurons, astrocytes, and oligodendrocytes.^104^ NSPCs have been observed to exist in the adult mammalian CNS.

However, the clinical effectiveness of endogenous neurogenesis in CNS repair is limited due to the susceptibility of neurons to certain environmental factors such as cytokines, oxygen, extracellular matrix, and chemokines. Following SCI, elevated levels of cytokines such as IL‐6 in the microenvironment can cause NSPCs to proliferate and differentiate exclusively into astrocytes rather than neurons. The insufficient number of endogenous NSPCs and/or damage to the original microenvironment after SCI may also contribute to this limited effectiveness.^105^

A study by Mikami et al.^106^ explored the interactions between immune cells and NSPCs through neutrosphere formation assays. The results demonstrated that co‐culturing NSPCs with DCs enhanced their survival and proliferation. Furthermore, the transplantation of DC into mice with damaged spinal cords activated the proliferation of endogenous neural stem cells and induced neurogenesis. These findings suggest that DCs can promote neural stem cell proliferation and survival by inducing NT‐3 production and activating endogenous microglia.

Additional research has shown that the NT‐3/TrKc‐mediated signaling pathway, which DCs activate, fosters the proliferation and differentiation of NSPCs.^107^ It is generally believed that exogenous NSPC transplantation can effectively replace lost or dysfunctional nerve cells in damaged areas. With exogenous NSPC transplantation, DCs can encourage neural stem cell differentiation into neurons and reduce astrocyte generation by releasing NT‐3 and other neurotrophic factors.^108^ Moreover, T cell‐induced neuroprotective responses promote recruitment, proliferation, and differentiation of endogenous neural stem and progenitor cells.^109^

## 6 CONCLUSION AND PERSPECTIVE

Current research suggests that DCs benefit SCI, mainly when activated by CNS antigens. As the most potent APC, DCs' therapeutic effects are T‐cell‐mediated. Essentially, DCs initiate the differentiation of T‐cells, activating microglia and astrocytes. Together, these cells secrete neurotrophic factors and regulate the microenvironment at the injury site, producing an anti‐inflammatory and pro‐repair effect. DCs also facilitate immunomodulation and immune tolerance, which can mitigate the harmful effects of inflammatory cells on tissues and cells. While this approach can potentially reduce secondary damage, further research is necessary to determine the specific mechanism.

After an SCI, the inflammatory response plays a vital role in developing secondary injuries. These injuries can impede recovery. Researchers have tried various strategies to regulate the inflammatory response to reduce these injuries and promote functional healing. These include employing immunosuppressants, immune cell inhibitors, chemokine receptor antagonists, and exogenous cytokines to control the microenvironment. Although some of these approaches have successfully reduced inflammation, they have not significantly improved limb function recovery. This might be because they do not promote neuron regeneration, which is essential for recovery. A comprehensive understanding of the inflammatory response and its complex interaction with other injury mechanisms is vital to identify new targets and develop more effective treatments.

Recent studies suggest that cells involved in the inflammatory response during SCI can either promote healing or cause further damage, depending on the stage of the injury. The ambiguity is likely due to the production of various inflammatory mediators or the strict regulation of the immune system. Initially, the cells and damaged tissue are targeted for clearance after SCI, but this process may also harm healthy cells. As time passes, the ability of inflammatory cells to promote repair becomes limited by the existing microenvironment, leading to an imbalance between the pro‐inflammatory and pro‐repair functions. Therefore, experts believe adjusting the immune response's balance may be more effective than directly eliminating its components. Further research is required to fully comprehend the dynamic changes and interactions of the immune system after SCI and to develop more individualized approaches to regulate the inflammatory response.

The application of DCs has demonstrated promising therapeutic effects in alleviating the damage‐repair function of inflammatory cells, thus presenting a potential research direction. DCs can activate a series of inflammatory cells, thereby producing a more significant number of neurotrophic factors than simply injecting trophic factors into the injury site. DCs, in conjunction with stem cell transplantation or other treatment modalities, hold great promise in regenerating functional neurons and axons and promoting high‐quality recovery. The potential of DCs to promote nerve regeneration and repair presents a compelling avenue for future research and development of SCI treatments. However, further investigation is required to determine the appropriate application of these findings in clinical treatment.

## AUTHOR CONTRIBUTIONS

Each author is expected to have made substantial contributions to the conception. Tian Cheng, Hongjian Liu, and Chao Jiang designed the work. Xiaonan Han, Mingkang Zhang, Liyan Yan, Yikun Fu, and Junmin Wang wrote the manuscript. Hongwei Kou and Chunfeng Shang created the figure of the manuscript. Jian Wang intellectually contributed and revised the manuscript. All the authors read and approved the manuscript.

## FUNDING INFORMATION

This work was supported by Henan Provincial Health Commission (SBGJ202302056) the National Natural Science Foundation of China (No. 82371339).

## CONFLICT OF INTEREST STATEMENT

The authors declare that they have no conflict of interest.

## Data Availability

This is a review; availability of data and materials is “not applicable.”
